# Gene expression divergence and nucleotide differentiation between males of different color morphs and mating strategies in the ruff

**DOI:** 10.1002/ece3.370

**Published:** 2012-08-31

**Authors:** Robert Ekblom, Lindsay L Farrell, David B Lank, Terry Burke

**Affiliations:** 1 Department of Ecology and Genetics, Evolutionary Biology Centre, Uppsala UniversityNorbyvägen 18 D, SE-75236, Uppsala, Sweden; 2 Department of Animal and Plant Sciences, University of SheffieldSheffield, S10 2TN, UK; 3 Department of Biological Sciences, Simon Fraser UniversityBurnaby, British Columbia, V5A 1S6, Canada

**Keywords:** Birds, genomics, lek, next generation sequencing, RNA-seq, ruff

## Abstract

By next generation transcriptome sequencing, it is possible to obtain data on both nucleotide sequence variation and gene expression. We have used this approach (RNA-Seq) to investigate the genetic basis for differences in plumage coloration and mating strategies in a non-model bird species, the ruff (*Philomachus pugnax*). Ruff males show enormous variation in the coloration of ornamental feathers, used for individual recognition. This polymorphism is linked to reproductive strategies, with dark males (Independents) defending territories on leks against other Independents, whereas white morphs (Satellites) co-occupy Independent's courts without agonistic interactions. Previous work found a strong genetic component for mating strategy, but the genes involved were not identified. We present feather transcriptome data of more than 6,000 de-novo sequenced ruff genes (although with limited coverage for many of them). None of the identified genes showed significant expression divergence between males, but many genetic markers showed nucleotide differentiation between different color morphs and mating strategies. These include several feather keratin genes, splicing factors, and the Xg blood-group gene. Many of the genes with significant genetic structure between mating strategies have not yet been annotated and their functions remain to be elucidated. We also conducted in-depth investigations of 28 pre-identified coloration candidate genes. Two of these (EDNRB and TYR) were specifically expressed in black- and rust-colored males, respectively. We have demonstrated the utility of next generation transcriptome sequencing for identifying and genotyping large number of genetic markers in a non-model species without previous genomic resources, and highlight the potential of this approach for addressing the genetic basis of ecologically important variation.

## Introduction

Understanding the genetic mechanisms controlling stable morphological or behavioral polymorphisms in natural population is currently a very active field of research (Stapley et al. 2010). Both nucleotide divergence and differences in expression rates of genes may be involved in such variation. For example, a recent study of horned beetles (*Onthophagus spp*.) found that patterns of gene expression in growing ornaments between two male morphs (that also represent different mating strategies) were as divergent as they were between males and females (Snell-Rood et al. 2011). Strong genetic differences (both nucleotide divergence and variation in gene expression rates) were also found between normal and dwarf morphs of lake whitefish (*Coregonus clupeaformis*) (Jeukens et al. 2010).

Plumage and coat color variation in vertebrates have evolved to function in multiple contexts, including thermoregulation, crypticity, signaling species, age, or individual identity, and indicating variation in individual quality (Dale 2006). In vertebrates, coloration is produced by two major classes of pigments, carotonoids, and melanains, as well as structural iridescence (Hill and McGraw 2006). Some structural and regulatory genes controlling the deposition of pigments have been identified. In the beach mouse (*Peromyscus polionotus*), for example, a mutation in the MC1R gene causes color pattern variation (Hoekstra et al. 2006); and in gray wolf (*Canis lupus*), a mutation in a beta-defensin gene is involved (Anderson et al. 2009). A natural color polymorphism (white or tan crown stripe) in the white-throated sparrow (*Zonotrichia albicollis*), which is also linked to dominance and mating strategies, is genetically determined through a dominant (for white) chromosomal polymorphism on autosome 2 (Watt et al. 1984; Tuttle 2003). This chromosomal inversion of almost 100 Mb has been studied in some detail, but the genes responsible for color and behavioral polymorphisms have yet not been identified (Thomas et al. 2008; Romanov et al. 2009). Rather simple genetic mechanisms for color polymorphisms and a link to behavior and reproductive strategies have also been found in a number of other birds (Roulin 2004) and other animals (McKinnon and Pierotti 2010). However, it remains largely unknown whether such correlated genetic polymorphisms arise due to shared regulatory mechanisms (such as variation in transcription factors influencing both traits), joint hormone pathways or physical linkage of different causative mutations. In the Soay sheep (*Ovis aries*), there is a clear genetic linkage between the gene responsible for coat color polymorphism (TYRP) and size (Gratten et al. 2008), indicating that several genetic polymorphisms important to fitness are co-localized in a small genomic region.

**Figure 1:**
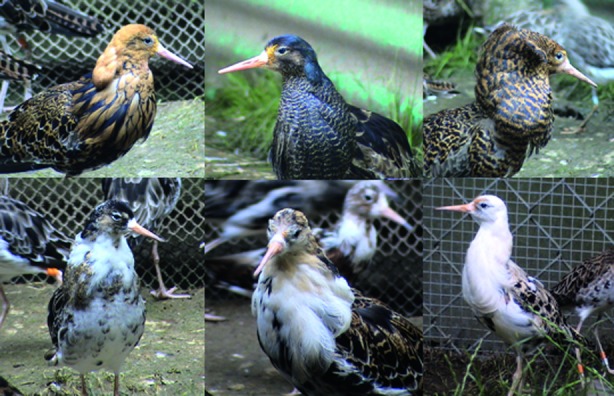
Typical male color and feather pattern variation in the ruff (*Philomachus pugnax*). All pictures are from the captive population used in this study. Color pattern and variation differs between individuals, between and within morph types in this species. The two rightmost individuals on the lower panel have the Satellite mating strategy and the rest of the males are Independents.

The ruff *Philomachus pugnax* is a lek-breeding shorebird with uniquely hyper-variable male breeding plumage colorations and patterns, which are permanent features of individuals (Fig. 1). Each spring, males grow elaborate ornamental neck ruffs and ear tufts, which vary independently from each other in color and pattern (Dale et al. 2001; Lank and Dale 2001). The background color within the ruff and tuft feathers is essentially white (no melanin), reddish brown (“rust,” phaeomelanin), or black (eumelanin). The individual ruff and head tuft feathers can be either plain or patterned with thick or thin bars or spots, and may be diluted. The ruff and head tufts as feather tracts may consist of uniform feather types or regular or irregular mosaics. This extensive and complex variation means that male ruffs within a lek are morphologically distinct, suggesting that plumage variation may be used in individual identification. Ruff mating displays are silent, and the plumage variation may have replaced the vocal signals commonly used by birds for this function (Lank and Dale 2001). Polymorphism in genes determining coloration has been proposed to be maintained by negative frequency selection in the ruff system. Given that a major function of the plumage polymorphism is to promote individual identification, novel mutations would provide more information used to distinguish individuals, and would thus be selected for (Dale et al. 2001).

Ranges of color variation in breeding plumage in ruff are tightly correlated with a fixed behavioral dimorphism in male mating strategy, which is strongly heritable and controlled by a simple Mendelian genetic polymorphism. An autosomal gene (the *S* locus) co-determines predominantly light versus dark coloration of the elaborate breeding plumage and male mating behavior (Lank et al. 1995, 1999). Males with substantial amounts of black in either their ruff or head tufts are “Independents,” which defend small lek-mating courts against other Independents. Males with predominantly white plumages behave as non-territorial “Satellites,” which form uneasy transient alliances with court holders. Co-occupied courts attract more females than do individual territorial males, stabilizing some level of cooperation between these reproductive competitors (van Rhijn 1991; Hugie and Lank 1997; Widemo 1998). Recently, a remarkable third “Faeder” morph was also described; these males resemble slightly large unadorned females morphologically and refrain from obvious male courtship displays, behaving as “sneakers” instead (Jukema and Piersma 2006).

The inheritance of ruff plumage variation has not yet been formally modeled, but the tight association between behavioral morph and coloration strongly suggests genetic determination. Also, inspection of pedigrees clearly shows a strong heritable component to coloration of the male ornamental feathers (D.B.L unpubl. data). Like other scolopacidae, feather colorations are produced by combinations of eumelanin and phaeomelanin. Only a few genes that affect plumage pigmentation or patterning in birds have so far been identified (Hubbard et al. 2010), and there are no obvious candidates for the ruff loci at present. The specific genetic mechanism that maintains the association between the behavioral strategy and plumage coloration for ornamented males is also unknown. Identifying the genetic region(s) involved would thus provide a novel mechanistic link between morphology and behavior variation (Bertossa 2010).

Next generation sequencing is currently revolutionizing the field of adaptation genetics (Stapley et al. 2010). With the novel techniques available, it is now possible to identify molecular markers on a genome-wide scale. Genomic scans for genes and markers segregating between morphs or populations can also be conducted at a very reasonable cost, even in non-model organisms without prior genomic information. One big advantage of this strategy, compared with previous genome scans (that relied on anonymous AFLP markers or microsatellites) is that the markers can be annotated and the genes or genetic regions with positive results can easily be identified, especially if the transcriptome (all expressed genes) is being sequenced (Wheat 2010; Ekblom and Galindo 2011). Such a transcriptomic approach, commonly known as RNA-Seq (Wang et al. 2009), can yield information, not only about nucleotide polymorphisms and genetic structure but also on differential gene expression levels (t Hoen et al. 2008).

The aim of this study was to identify genetic regions that might be involved in determining variation in color morph and mating strategies in males of the ruff. We have used 454 sequencing to characterize the transcriptome from feather samples of several individuals in a captive ruff population, and use these data to investigate both gene expression level divergence and nucleotide sequence differentiation between different males. The genes identified here as potential candidates for regulating color and mating strategy polymorphism can be investigated in more depth in the future using a candidate gene approach. To this end, we also identify several hundred Single Nucleotide Polymorphisms (SNPs) and microsatellites from the ruff transcriptome, markers that can be used in follow-up studies of this intriguing study species.

## Materials and Methods

### Study population, behavior observations, and feather sampling

**Figure 2:**
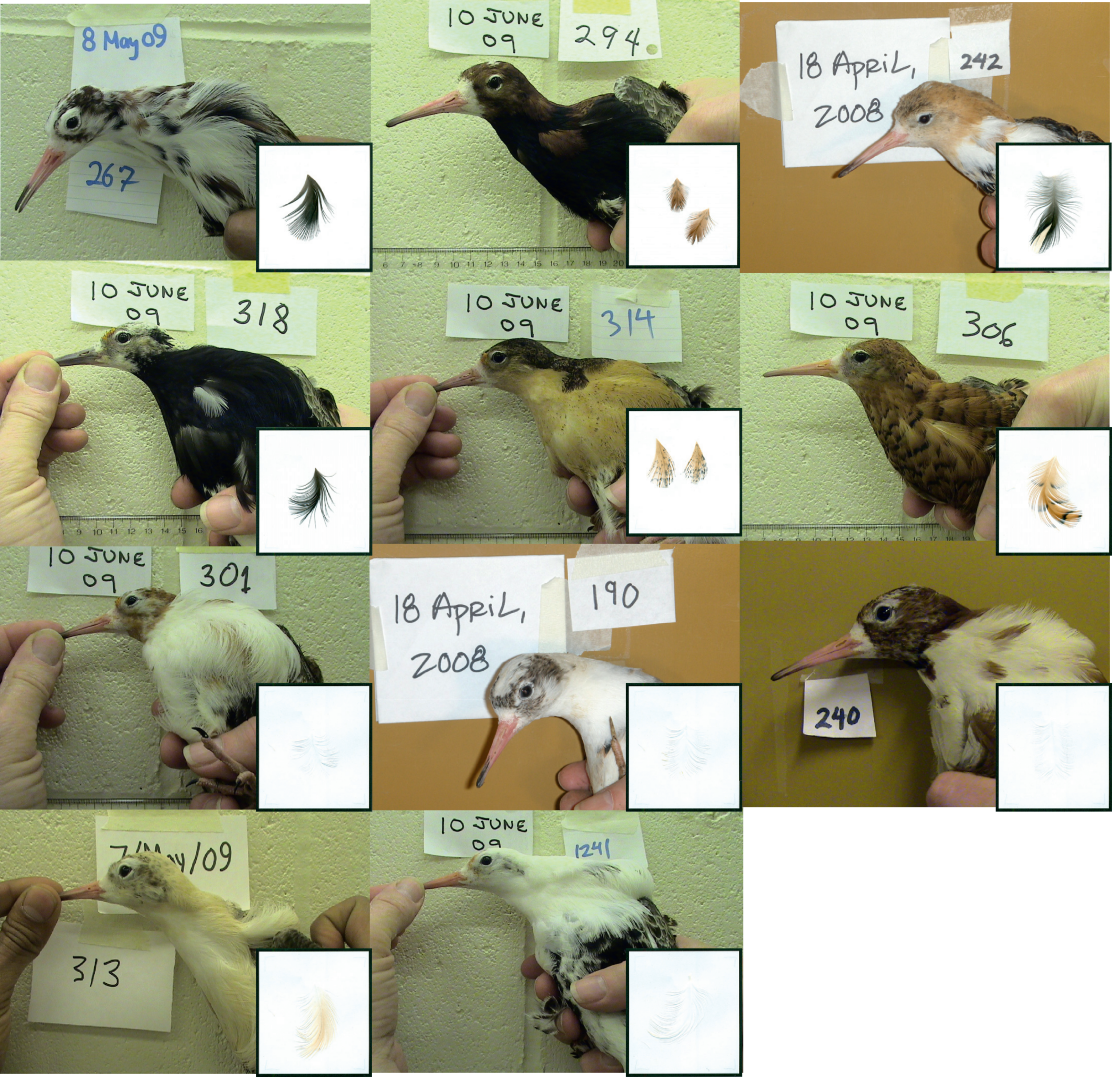
Photographs of the male ruffs and sampled feathers (inserted frames) used in this study. Clockwise numbering: 267, 294, 242, 318, 314, 306, 301, 190, 240, 313, 1241.

Actively growing ornamental feathers were plucked from 11 males in a captive breeding population of ruffs maintained by DBL, and immediately placed into RNAlater (Table 1, Fig. 2). The founders of the captive breeding flock were 56 males and 64 females hatched from eggs collected near Oulu Finland in 1985, 1989, and 1990, and bred continuously thereafter. Population size has varied from 34 in 1985 to ca. 175–200 in 2009 when feathers were sampled. The feathers sampled were classified as: black, rust, or white; two were patterned. Black ruff feathers are typically iridescent, produced by keratin structure. As we were interested in the genes active when the feather was plucked, we classified feathers based on the color growing at the time they were plucked; two feathers with patterns at the tip were called uniform because the growing lower part of the feather were solid. The behavioral morphs of males as Independents or Satellites were known from behavioral observations in previous years.

**Table 1:** Sequence data on each sample separately, Mean length of reads are shown within brackets. Information on color morph and mating strategy of the individual males is also provided. Individual 313 with color “Straw” was included in the “Rust” category in the analyses of color pattern, whereas individuals 294 and 306, which have multiple colors (barred or flecked), were included both in the “Black” and in the “Rust” categories

Individual	No raw reads	No trimmed reads	Color	Mating strategy
267	25,178 (275)	19,909 (292)	Black	Satellite
242	24,823 (276)	19,714 (293)	Black	Independent
294	17,508 (271)	13,163 (298)	Rust/black	Independent
318	37,927 (277)	30,017 (296)	Black	Independent
314	28,362 (272)	21,390 (297)	Rust	Independent
306	33,297 (278)	26,190 (296)	Rust/black	Independent
301	24,563 (270)	18,987 (293)	White	Satellite
190	37,743 (263)	28,176 (294)	White	Satellite
240	43,835 (273)	33,462 (299)	White	Satellite
313	30,617 (296)	25,596 (307)	Straw	Satellite
1241	46,759 (288)	37,721 (305)	White	Satellite
Total	350,648 (277)	274,325 (297)		

### cDNA library preparation and sequencing

Total RNA was extracted from the feather samples using the miRvana kit (Ambion, Carlsbad, California). The RNA was treated with TURBO DNase (Ambion) to eliminate any contamination of gDNA. Synthesis of cDNA was performed using the SMART kit (Clontech, Saint-German-en-Laye, France), following the manufacturers' protocol and using 22 cycles for the final amplification step. Each cDNA library was individually tagged and sequenced on half a plate of a 454 Genome Sequences FLX system (Roche, Branford, Conneticut) at the sequencing facility in Liverpool (School of Biological Sciences, University of Liverpool). All raw 454-sequencing reads are available through the GenBank/SRA database under accession number SRA049313.1.

### Assembly and annotation

A total of 350,648 sequence reads were produced from the 11 males. After trimming away bad quality, SMART primer and poly A sequence, using NGen 2.0 (DNASTAR Inc. Madison, Wisconsin), 274,325 reads with a mean read length of 297 nucleotides remained (see Table 1 for information about number and length of reads for each individual separately). De-novo assembly of trimmed reads (from all individuals together) was performed using NGen 2.0 (DNASTAR Inc.) using parameters specified in Ekblom et al. (2010). The sequences of all contigs produced are available upon request from the corresponding author. Mapped assemblies for sequence data from each individual separately were also performed using the contigs from the de-novo assembly as a template.

All contigs and singletons were annotated using a blast approach. The contig and singleton sequences were compared with the chicken protein database (WASHUC2.56, downloaded from the ENSEMBL ftp site; http://www.ensembl.org/info/data/ftp/index.html) using stand alone version 2.2.18 of blastx (Altschul et al. 1997). Only the best blast hit for each query sequence was kept and only hits with an e-value below 10^−5^, and where the difference in e-values between the best blast hit and the second best hit was at least one order of magnitude. For contigs with SNPs (see below) that could not be annotated from the chicken protein database, we also blasted sequences against zebra finch gene predictions, human protein database, and the nr (non-redundant) sequence database using the web blast interface of NCBI (http://blast.ncbi.nlm.nih.gov/Blast.cgi).

### Expression divergence

The level of transcription for each gene and individual was measured by counting up the number of reads from that individual that mapped to the gene in question. Expression analyses were performed using the bioconductor, edgeR package (Robinson et al. 2010), using a common dispersion parameter and manually adjusting the library size to the number of trimmed reads entering the templated assembly. Differential expression of genes between different color morphs and mating strategies were tested using an exact test for the negative binomial distribution and applying false discovery rate (FDR) correction (Benjamini and Hochberg 1995).

### Nucleotide differentiation

Single nucleotide polymorphisms were identified from the ruff transcriptome using the PanGEA software (Kofler et al. 2009), using mostly default settings. First, all the reads were mapped to the contigs using the homopolymer Smith–Waterman algorithm. Then, a de-novo SNP identification was performed using the “454 SNP-identification mode” and allowing only one SNP (the one with highest coverage) per contig, no indels, at least 10 sequences coverage at the SNP site, a minimum of two reads with the minor allele and a maximum of two alleles at a given site. We only used one SNP per contig to avoid problems of pseudo-replication due to linkage of closely situated markers, a minimum of 10 reads per SNP to be able to confidently score genotypes and a minimum of two reads with the minor allele to account for sequencing errors. All individuals where both alleles occurred were considered to be heterozygotes, whereas all individuals where only one of the alleles was found were scored as homozygotes. For analyses of nucleotide differentiation, only SNPs that were scored in at least nine individuals were considered. One hundred nucleotides upstream and downstream of the SNP (or to the end of the contig sequence if less than 100 nucleotides) were extracted from the consensus contig sequences. These were then mapped onto the chicken genome sequence (using blastn) to assess the genomic locations of the SNPs. In general, there are high levels of synteny even between quite divergent bird species like the chicken and the zebra finch (Warren et al. 2010), and the location of the marker in the chicken genome can thus be used as an estimation of the location in the ruff as well.

Tests for genetic differentiation between groups of individuals with different color and mating strategy were performed using the “population differentiation” option in GenePop (http://genepop.curtin.edu.au/) (Raymond and Rousset 1995) testing for both genic differentiation (differences in allele frequencies) and genotypic differentiation (differences in allele combinations). Test for genotypic differentiation was also conducted using the *G*-test implemented in the SAM software (Joost et al. 2008). LOSITAN (Antao et al. 2008) was used to conduct an *F*_ST_ outlier analysis, testing each loci for deviations in structure (between color morphs and mating strategies) from neutral expectations of the relationship between heterozygosity and *F*_ST_. Positive selection was inferred from the LOSITAN analysis if the given *P*-value was higher than 0.975 and the estimated *F*_ST_ was above 0.25.

### Coloration candidate gene annotation

We searched the literature for genes involved in bird coloration and/or territorial aggression and compiled a list of 28 candidates (Appendix A1). The 454-sequence blast results were then manually searched for these genes using the Ensembl chicken protein identifiers. We extracted both information of expression of the candidate genes and sequence data from contigs and singletons mapping to the genes in question and used this in manual annotation of these genes. We also aligned all reads and contigs mapping to a candidate gene with the chicken coding sequence for the gene (downloaded from ENSEMBL BioMart: http://www.ensembl.org/biomart) using the ClustalW algorithm (Thompson et al. 1994).

### Microsatellite identification

Microsatellites were identified from the transcriptome 454-sequence data (both contigs and singletons) using the program MsatCommander (Faircloth 2008). We used a minimum number of 10 repeats for di-nucleotide repeats, 8 repeats for tri-nucleotides, and 4 nucleotides for tetra-, penta-, and hexa-nucleotides.

## Results

### Sequencing and assembly

**Figure 3:**
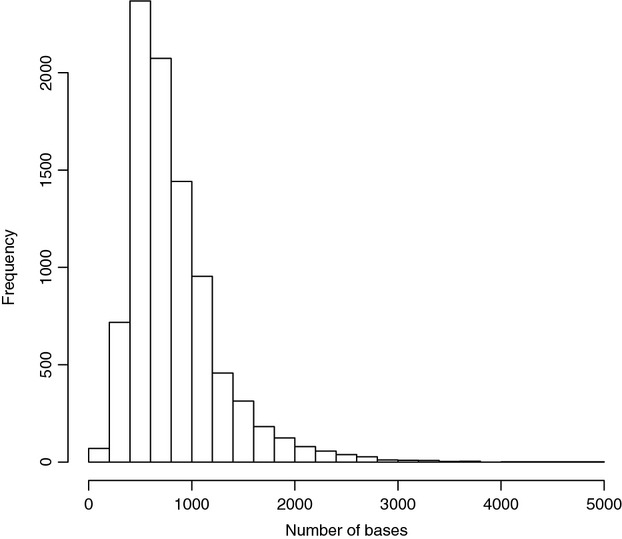
Distribution of the length (base pairs) of assembled contigs of the ruff feather transcriptome. Sequence data from 11 different males were assembled together.

**Figure 4:**
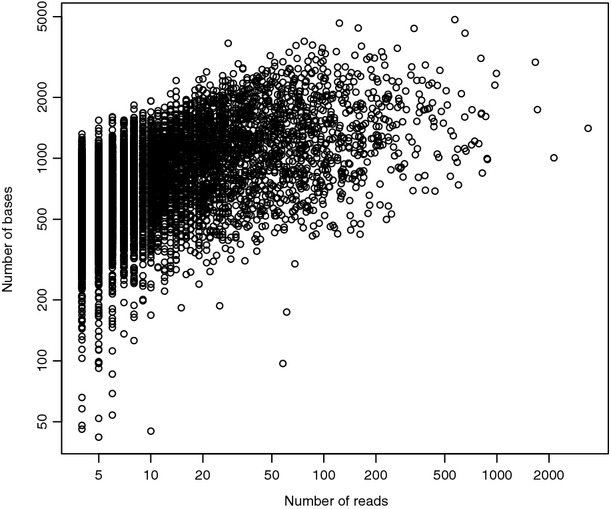
Relationship between contig depth (number of reads) and length (number of base pairs) for the ruff feather transcriptome.

cDNA libraries from 11 different ruff males were sequenced using the 454 technology (Table 1). A total of 274,325 reads remained after trimming. Of these, 193,929 (70%) assembled into 8943 contigs with a mean contig length of 828 nucleotides (range 42–4837) and a mean of 21.7 reads per contig (Fig. 3). As expected, there was a strong positive correlation between (log) contig length and (log) contig depth (*r*_p_ = 0.55, df = 8941, *P* < 0.0001; Fig. 4).

We were able to identify 6309 genes in the Ruff transcriptome by blasting the contigs and singletons against the chicken protein database. This represents about one-fourth of all currently annotated chicken genes, but most of these were only partly covered with sequence reads (the mean length of chicken genes are around 1800 base pairs while our mean contig size was only 828 base pairs). Note, however, that several contigs may map to different parts of the same gene. A total of 371 genes were present in the transcripts of all 11 sampled individuals, while 2286 genes were present in the transcripts of one individual only.

### Expression divergence

**Figure 5:**
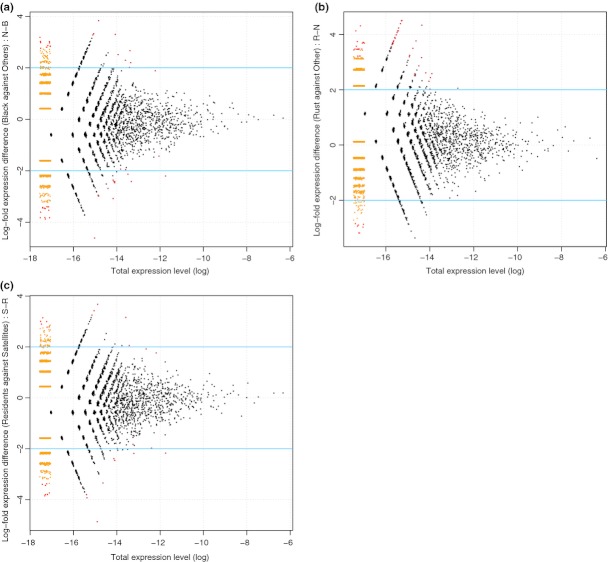
Plot of expression divergence between (a) black males and males of other colors, (b) rust-colored males compared with males with different colors and (c) Independent compared with Satellite males. Genes more expressed in black (a), rust (b), and Independent (c) males have negative log-fold expression levels. The blue horizontal lines represent four-fold differences in expression between morphs. Genes that are only expressed in one of the morphs are plotted in yellow smears to the left in the graph. Red points represent genes with expression divergence between morphs (*P* < 0.01, without multiple test correction). None of the genes were significantly differentially expressed after applying false discovery rate correction.

The differential expression analysis revealed 22 genes that were up-regulated and 20 down-regulated (unadjusted *P* < 0.01) in black males compared with others (Fig. 5a). In rust-colored individuals, 7 genes were found to be up-regulated and 50 down-regulated compared with individuals with other colors (Fig. 5b). Six genes had higher expression in Satellite males compared with Independents and 25 had higher expression in Independents (Fig. 5c). However, none of these expression differences between male mating strategies remained statistically significant after multiple test correction (*P* > 0.05).

Out of the 28 pre-identified coloration candidate genes (Appendix S2), 11 had observed expression in the feather tissue. However, most of these had very low levels of transcription with only one or a few reads present in the dataset. The Endothelin receptor B gene (EDNRB; known to be involved in mice coat color variation) was expressed in two uniformly black males (out of three), but was completely absent from all eight differently colored males. This is suggestive of differential expression in pure black morphs, but as expression was so low, this could not be verified using the expression divergence test. The gene coding for tyrosinase (TYR; coding for an important catalytic enzyme in the melanin synthesis pathway) was represented by five reads in two rust-colored males (out of four), while none of the other males showed expression of this gene. This expression difference was actually significant (*P* = 0.0039), but did not remain significant after the false discovery rate correction in the test across all transcripts.

### Nucleotide differentiation

**Figure 6:**
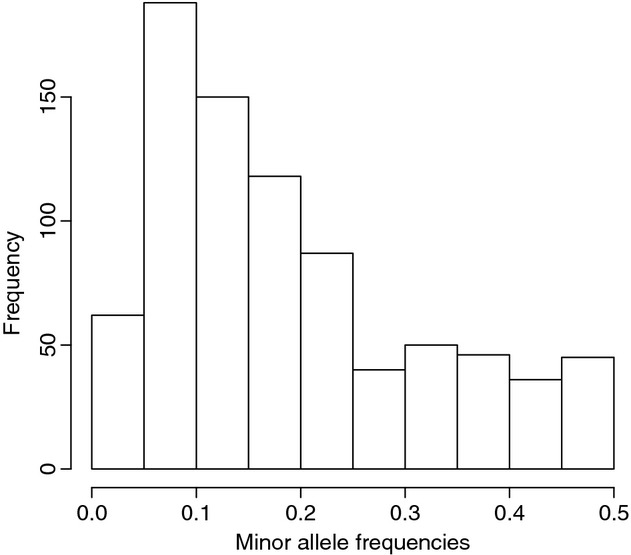
Distribution of minor allele frequencies for the 822 SNP markers identified from the feather transcriptome of 11 ruff males.

SNPs were identified in 822 of the 8943 contigs (only one SNP per contig and only sites with a depth of more than 10 reads were considered, see methods; Appendix A2, A3). These polymorphisms represented 681 transitions and 141 transversions, and the GC content at the SNP sites were 50.67%. Minor allele frequencies (MAF) ranged between 0.045 and 0.5 (mean = 0.20; Fig. 6). Three hundred and sixty-six of the SNPs could be scored in at least nine individuals (mean MAF = 0.18) and were used for nucleotide differentiation analyses.

**Figure 7:**
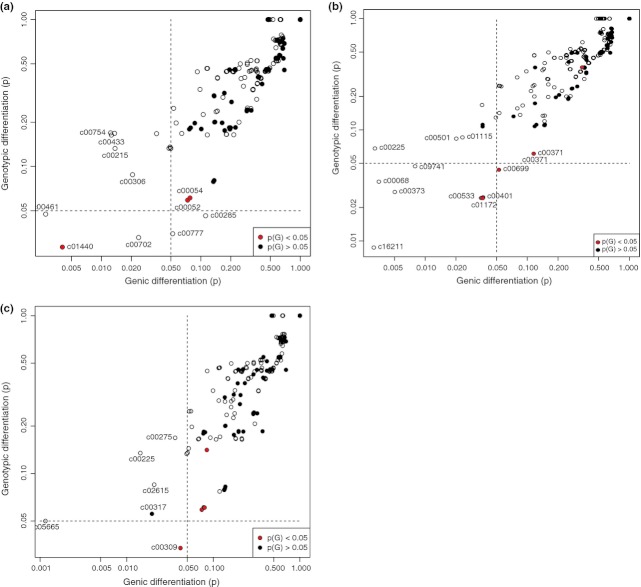
Outputted *P*-values from the GenePop analysis testing for nucleotide differentiation between (a) black males and males of other colors, (b) rust-colored males compared with males with different colors, and (c) Independent compared with Satellite males. Test for differences in allele frequencies (genic differentiation) is shown on the *X*-axis while test for differences in haplotype frequencies (genotypic differentiation) is shown on the *Y*-axis. Dashed lines indicate significance thresholds of *P* = 0.05. Markers with significant structure inferred from the G-test in the SAM software are highlighted in red. The names of some of the most differentiated markers (see Tables 2–4) are given in the figures.

Fifteen SNP markers were significantly differentiated between black and non-black individuals for either allele or genotype frequencies using GenePop (Raymond and Rousset 1995) (Fig. 7a, Table 2). One of these (c01440) was also identified as having significant structure using SAM (Joost et al. 2008). This marker is located in the Xg blood-group gene. Two SNPs with significant structure between black and non-black males were located in feather keratin genes (c00285 and c00777).

**Table 2:** SNP markers identified as being genetically structured between black and non-black ruff males. “Allelep” and “genotypep” are outputted P-values from GenePop using the gene and genotype option. “G” and the p value for G (pG) are calculated from the SAM software. Heterozygosity (Het), *F*_ST_ (*F*st), and *P* were calculated in LOSITAN and markers with pos.sel = 1 were identified as being under positive selection with this software. Markers highlighted with bold text were identified as structured using at least two of these independent tests

Locus	Allelep	genotypep	G	pG	Het	Fst	P	pos.sel	Annotation	Gene description
c00052	0.07	0.06	6.16	0.05	0.30	0.22	0.88	0	ENSTGUT00000003716	microtubule-associated protein 1 light chain 3 alpha-like
c00054	0.08	0.06	6.16	0.05	0.30	0.22	0.88	0	ENSGALP00000030189	cytochrome c oxidase subunit Vic
**c00215**	**0.01**	**0.13**	**NA**	**NA**	**0.50**	**0.43**	**1.00**	**1**	**gi**^**|**^**118100421**^**|**^**ref**^**|**^**XM_415902.2**^**|**^	**DUSP14 dual specificity phosphatase 14**
**c00275**	**0.04**	**0.17**	**NA**	**NA**	**0.50**	**0.44**	**1.00**	**1**	**ENSGALP00000028033**	**Novel protein coding**
c00279	0.06	0.20	NA	NA	0.42	0.36	1.00	1	ENSGALP00000018498	Putative uncharacterized protein
c00285	0.11	0.05	NA	NA	0.50	0.26	0.96	0	ENSGALP00000006118	keratin 10
**c00306**	**0.02**	**0.09**	**NA**	**NA**	**0.66**	**0.49**	**0.99**	**1**	**ENSTGUT00000007814**	**DEP domain-containing protein 6**
c00355	0.05	0.14	NA	NA	0.38	0.29	0.94	0	gi^|^226823205^|^ref^|^NM_001159347.1^|^	neuron navigator 3
c00358	0.05	0.13	NA	NA	0.38	0.29	0.94	0	ENSGALP00000002235	stathmin 1
**c00433**	**0.01**	**0.16**	**NA**	**NA**	**0.60**	**0.56**	**1.00**	**1**	**ENSGALP00000015448**	**similar to neuropeptide Y receptor Y5**
**c00461**	**0.00**	**0.05**	**NA**	**NA**	**0.70**	**0.67**	**1.00**	**1**	**ENSGALP00000038287**	**calponin 3, acidic (cytoskeleton)**
c00633	0.09	0.44	NA	NA	0.40	0.33	0.98	1	ENSGALP00000023115	FK506 binding protein 4, 59kDa
c00648	0.05	0.13	NA	NA	0.38	0.29	0.94	0	ENSTGUT00000008828	Protein Shroom2 (Apical-like protein)
**c00702**	**0.02**	**0.03**	**NA**	**NA**	**0.65**	**0.45**	**0.99**	**1**	**–**	**–**
**c00754**	**0.01**	**0.17**	**NA**	**NA**	**0.60**	**0.56**	**1.00**	**1**	**ENSTGUT00000002899**	**hypothetical protein LOC100219861**
c00777	0.05	0.03	NA	NA	0.38	0.29	0.94	0	ENSGALP00000029159	similar to Scale keratin (S-ker)
**c01440**	**0.00**	**0.03**	**6.78**	**0.03**	**0.70**	**0.56**	**1.00**	**1**	**ENSGALP00000026834**	**Xg blood group**
c01504	0.05	0.13	NA	NA	0.38	0.29	0.94	0	ENSTGUT00000000045	Lon protease homolog, mitochondrial Precursor
c01511	0.05	0.25	NA	NA	0.50	0.45	1.00	1	–	–
c03886	0.06	0.17	NA	NA	0.58	0.32	0.98	1	ENSTGUT00000008476	Tyrosine-protein kinase Tec
**c05665**	**0.01**	**0.17**	**NA**	**NA**	**0.60**	**0.56**	**1.00**	**1**	**gi**^**|**^**47021392**^**|**^**emb**^**|**^**CR405874.1**^**|**^	**Gallus gallus finished cDNA, clone ChEST575e12**

Comparing rust-colored individuals against other individuals, there were 15 significantly differentiated markers (Fig. 7b, Table 3). Four of these were also structured according to the SAM analysis. Several of the differentiated markers (c00401, c00533, c1172, c16211) were found to be situated in the same gene; “similar to type 1 hair keratin KA31.” These markers were all heterozygous in most of the rust-colored individuals, whereas homozygous for the major allele in males with other coloration. Two other keratin genes (“Feather keratin 2” and “similar to feather keratin”) were also among the differentiated markers.

**Table 3:** SNP markers identified as being genetically structured between rust colored and other ruff males. “allelep” and “genotypep” are outputted p values from GenePop using the gene and genotype option. “G” and the p value for G (pG) are calculated from the SAM software. Heterozygosity (Het), *F*_ST_ (*F*st), and *P* were calculated in LOSITAN and markers with pos.sel = 1 were identified as being under positive selection with this software. Markers highlighted with bold text were identified as structured using at least two of these independent tests

Locus	Allelep	Genotypep	G	pG	Het	Fst	P	pos.sel	Annotation	Gene description
c00018	0.15	0.11	4.86	0.09	0.29	0.23	1.00	1	ENSTGUT00000001822	cytoplasmic linker associated protein 2
c00031	0.04	0.11	4.89	0.09	0.38	0.29	0.96	0	ENSGALP00000023950	GJA1 gap junction protein, alpha 1
c00053	0.15	0.11	4.86	0.09	0.29	0.23	1.00	1	ENSGALP00000019543	cytoplasmic linker associated protein 2
**c00068**	**0.00**	**0.03**	**NA**	**NA**	**0.63**	**0.57**	**0.99**	**1**	**gi**^**|**^**45424070**^**|**^**emb**^**|**^**CR353181.1**^**|**^	**Gallus gallus finished cDNA, clone ChEST441h23**
c00153	0.27	0.42	NA	NA	0.29	0.23	1.00	1	gi^|^46429612^|^emb^|^CR390967.1^|^	Gallus gallus finished cDNA, clone ChEST542i20
**c00225**	**0.00**	**0.07**	**NA**	**NA**	**0.67**	**0.60**	**1.00**	**1**	**ENSGALP00000012295**	**Gallus gallus hypothetical LOC428049**
**c00275**	**0.04**	**0.17**	**NA**	**NA**	**0.50**	**0.44**	**1.00**	**1**	**ENSGALP00000028033**	**Novel gene**
c00279	0.15	0.35	NA	NA	0.36	0.31	0.98	1	ENSGALP00000018498	Putative uncharacterized protein
c00291	0.27	0.42	NA	NA	0.29	0.23	1.00	1	ENSGALP00000039989	similar to Scale keratin (S-ker)
c00328	0.09	0.17	NA	NA	0.40	0.33	0.99	1	gi^|^71897262^|^ref^|^NM_001030907.1	RCAN family member 3
c00371	0.12	0.06	9.42	0.01	0.50	0.29	0.95	0	ENSGALP00000010447	S-phase kinase-associated protein 1
**c00373**	**0.01**	**0.03**	**NA**	**NA**	**0.75**	**0.67**	**1.00**	**1**	**gi**^**|**^**296785148**^**|**^**gb**^**|**^**AC239375.3**^**|**^	**Chlorocebus aethiops BAC clone CH252-485N20**
**c00401**	**0.04**	**0.02**	**8.39**	**0.02**	**0.38**	**0.29**	**0.96**	**0**	**ENSGALP00000028174**	**similar to type I hair keratin KA31**
**c00501**	**0.02**	**0.08**	**NA**	**NA**	**0.69**	**0.52**	**0.99**	**1**	**ENSGALP00000000205**	**Ribosomal component**
**c00533**	**0.04**	**0.02**	**8.39**	**0.02**	**0.38**	**0.29**	**0.96**	**0**	**ENSGALP00000028174**	**similar to type I hair keratin KA31**
c00558	0.04	0.11	4.89	0.09	0.38	0.29	0.96	0	ENSGALP00000023266	ribosomal protein S20
c00692	0.15	0.11	4.86	0.09	0.29	0.23	1.00	1	ENSTGUT00000014027	39S ribosomal protein L48
**c00699**	**0.05**	**0.04**	**6.78**	**0.03**	**0.59**	**0.32**	**0.96**	**0**	**ENSGALP00000029198**	**Feather keratin 2**
c00754	0.05	0.25	NA	NA	0.50	0.45	1.00	1	ENSTGUT00000002899	hypothetical protein LOC100219861
c00760	0.35	0.37	6.78	0.03	0.50	0.07	0.74	0	ENSGALP00000007502	ATP synthase, H+ transporting, mitochondrial F0 complex (F2)
c00813	0.21	0.44	NA	NA	0.30	0.22	1.00	1	ENSTGUT00000007149	Phosducin-like protein (PHLP)
c00895	0.06	0.25	NA	NA	0.50	0.45	1.00	1	ENSTGUT00000017859	Novel gene
**c01115**	**0.02**	**0.09**	**NA**	**NA**	**0.50**	**0.40**	**0.99**	**1**	**ENSGALP00000028467**	**cold inducible RNA binding protein**
c01159	0.27	0.20	NA	NA	0.29	0.23	1.00	1	ENSTGUT00000006904	Histone acetyltransferase MYST4
**c01172**	**0.04**	**0.02**	**8.39**	**0.02**	**0.38**	**0.29**	**0.96**	**0**	**ENSGALP00000028174**	**similar to type I hair keratin KA31**
c01504	0.05	0.13	NA	NA	0.38	0.29	0.96	0	ENSTGUT00000000045	Lon protease homolog
c01558	0.35	0.61	NA	NA	0.29	0.23	1.00	1	ENSTGUT00000009454	Programmed cell death protein 5
c01802	0.05	0.25	NA	NA	0.50	0.45	1.00	1	ENSGALP00000001690	similar to feather keratin
**c09741**	**0.01**	**0.05**	**NA**	**NA**	**0.63**	**0.57**	**0.99**	**1**	**ENSTGUT00000000535**	**Protocadherin-24 Precursor**
c12157	0.05	0.14	NA	NA	0.50	0.45	1.00	1	ENSGALP00000012295	Putative uncharacterized protein VTGIII
**c16211**	**0.00**	**0.01**	**NA**	**NA**	**0.67**	**0.60**	**1.00**	**1**	**ENSGALP00000028174**	**similar to type I hair keratin KA31**
c18140	0.26	0.53	NA	NA	0.29	0.23	1.00	1	ENSGALP00000011539	Non-histone chromosomal protein HMG-14A

Seven SNPs were significantly differentiated between Independent and Satellite males (Fig. 7c, Table 4). Five markers were also found to have structure between these mating strategies using the SAM software, but only one of these overlapped with the GenePop results. Several of the structured SNPs were matching to un-annotated genes (c00255, c00275) or chicken cDNA clones of unknown origin (c00063, c05665).

**Table 4:** SNP markers identified as being genetically structured between ruff males with Independent and Satellite mating strategies. “allelep” and “genotypep” are outputted p values from GenePop using the gene and genotype option. “G” and the *P*-value for G (pG) are calculated from the SAM software. Heterozygosity (Het), *F*_ST_ (*F*st), and *P* were calculated in LOSITAN and markers with pos.sel = 1 were identified as being under positive selection with this software. Markers highlighted with bold text were identified as structured using at least two of these independent tests

Locus	Allelep	Genotypep	G	pG	Het	Fst	P	pos.sel	Annotation	Gene description
c00052	0.07	0.06	6.16	0.05	0.30	0.22	0.92	0	ENSTGUT00000003716	Microtubule-associated proteins 1A/1B light chain 3A Precursor
c00054	0.08	0.06	6.16	0.05	0.30	0.22	0.92	0	ENSGALP00000030189	cytochrome c oxidase subunit Vic
c00063	0.08	0.06	6.16	0.05	0.30	0.22	0.92	0	gi^|^46429612^|^emb^|^CR390967.1^|^	chicken cDNA clone
**c00225**	**0.01**	**0.13**	**NA**	**NA**	**0.50**	**0.43**	**1.00**	**1**	**ENSGALP00000012295**	**Q90811_CHICK**
**c00275**	**0.04**	**0.17**	**NA**	**NA**	**0.50**	**0.44**	**1.00**	**1**	**ENSGALP00000028033**	**Novel protein coding**
c00279	0.06	0.20	NA	NA	0.42	0.36	0.99	1	ENSGALP00000018498	Putative uncharacterized protein
**c00309**	**0.04**	**0.03**	**6.16**	**0.05**	**0.60**	**0.30**	**0.94**	**0**	**gi**^**|**^**71897266**^**|**^**ref**^**|**^**NM_001030906.1**^**|**^	**splicing factor, arginine/serine-rich 13A**
**c00317**	**0.02**	**0.06**	**4.75**	**0.09**	**0.58**	**0.40**	**0.99**	**1**	**ENSGALP00000002960**	**dynein, light chain, roadblock-type 1**
c00358	0.05	0.13	NA	NA	0.38	0.29	0.92	0	ENSGALP00000002235	Stathmin
c00433	0.06	0.25	NA	NA	0.50	0.45	1.00	1	ENSGALP00000015448	similar to neuropeptide Y receptor Y5
c00465	0.12	0.47	NA	NA	0.33	0.27	0.99	1	ENSGALP00000015687	Collagen alpha-2(I) chain
c00754	0.05	0.25	NA	NA	0.50	0.45	1.00	1	ENSTGUT00000002899	hypothetical protein LOC100219861
c00880	0.08	0.14	6.16	0.05	0.60	0.27	0.92	0	ENSGALP00000000205	Ribosomal structure protein
c01021	0.12	0.47	NA	NA	0.33	0.27	0.99	1	ENSGALP00000016629	Peroxiredoxin-1
c01159	0.12	0.08	NA	NA	0.33	0.27	0.99	1	ENSTGUT00000006904	Histone acetyltransferase MYST4
c02176	0.05	0.14	NA	NA	0.50	0.45	1.00	1	ENSTGUT00000003357	chromodomain helicase DNA binding protein 6
**c02615**	**0.02**	**0.09**	**NA**	**NA**	**0.64**	**0.45**	**0.98**	**1**	**–**	**–**
c05497	0.25	0.50	NA	NA	0.33	0.27	0.99	1	gi^|^61098492^|^gb^|^AC147215.4^|^	s-adenosylmethionine decarboxylase proenzyme 2-like
**c05665**	**0.00**	**0.05**	**NA**	**NA**	**0.75**	**0.71**	**1.00**	**1**	**gi**^**|**^**47021392**^**|**^**emb**^**|**^**CR405874.1**^**|**^	**Gallus gallus finished cDNA, clone ChEST575e12**
c18140	0.12	0.47	NA	NA	0.33	0.27	0.99	1	ENSGALP00000011539	Non-histone chromosomal protein HMG-14A

### *F*_ST_ outlier analysis

**Figure 8:**
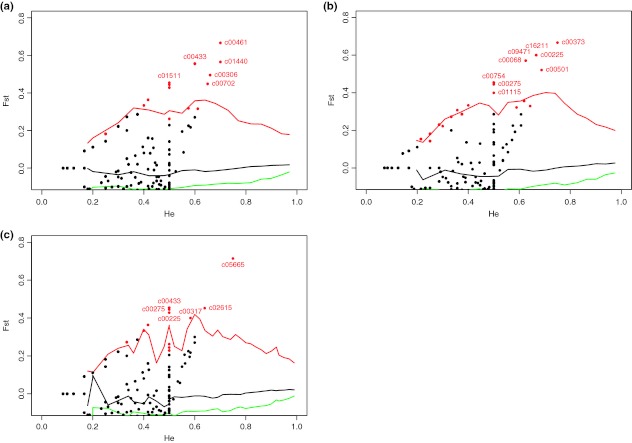
Output from *F*_ST_ outlier analyses performed in LOSITAN testing for structure between (a) black males and males of other colors, (b) rust-colored males compared with males with different colors, and (c) Independent compared with Satellite males. Black line represents mean *F*_ST_ for each given level of heterozygosity (He) and the red and green lines correspond to the 95% confidence interval thresholds of neutral expectations. Red points in the graphs represent candidates for positive selection (higher differentiation than expected by chance) and names of some of the strongest candidates are given in red labels. Note that some markers have identical *F*_ST_ and He, and are thus overlapping in the graphs.

Another way of investigating genetic structure between morphs is the *F*_ST_ outlier approach. We performed such an analysis using the software LOSITAN (Antao et al. 2008). Here, 13 SNPs were identified as having higher structure between black and non-black morphs compared with neutral expectations based on heterozygosity (Fig. 8a, Table 2). This is an indication of positive selection acting on the genes where these are situated (or closely linked genes). Nine of these overlapped with the markers were identified using the GenePop approach (Table 2). For rust-colored individuals, there were 23 *F*_ST_ outliers, of which eight were overlapping with the genetic structure analyses (Fig. 8b, Table 3). Finally, for males with the Independent mating strategy compared with Satellite males, there were 14 LOSITAN outliers, five of which overlapped with PopGen/SAM results (Fig. 8c, Table 4).

There was no correlation between gene-wise nucleotide differentiation and expression divergence between color morphs (black vs. others, *P*_genic differentiation_ against log-fold difference in expression: *r*_s_ = 0.107, df = 186, *P* = 0.145; rust vs. non-rust, *r*_s_ = 0.09, df = 186, *P* = 0.902) or between different mating strategies (Independents vs. Satellites: *r*_s_ = 0.072, df = 186, *P* = 0.3299). No SNPs were identified in any of the 28 pre-identified coloration candidate genes.

### Microsatellite identification

A total of 567 microsatellites were identified from the contigs and singletons, but only 199 had sufficient flanking sequence information to allow for primer design (Appendix A4). The most common type of microsatellite was tetra-nucleotide repeats with 252 identified loci; the least common was hexa-nucleotide repeats with only 28 loci (Table 5).

**Table 5:** Microsatellite repeats identified from the ruff transcriptome sequencing

Repeat type	Minimum repeat number	Total number	With primer sequence
Di	>10	157	27
Tri	>8	54	17
Tetra	>4	252	119
Penta	>4	76	35
Hexa	>4	28	1
Total		567	199

## Discussion

We have successfully sequenced, assembled, and annotated the feather transcriptome from a non-model bird species, the ruff, where there was previously very limited genomic data or tools available. By acquiring transcriptome sequence data separately from 11 different males, we were able to analyze differential gene expression as well as genetic sequence divergence between different color morphs and mating strategies of this lekking bird. Most studies that have been characterizing transcriptomes in non-model organisms so far have been very descriptive (Vera et al. 2008; Ekblom and Galindo 2011), addressing for example, tissue specific gene expression (Ekblom et al. 2010). A few studies have also used this approach to investigate genomics of speciation and adaptation by comparing transcriptome libraries between different subspecies or ecotypes of the same species (Galindo et al. 2010; Hohenlohe et al. 2010a; Wolf et al. 2010). For example, Goetz et al. (2010) used an approach very similar to ours, to address genetic bases for morphological differentiation between two forms of lake trout (*Salvelinus namaycush*). They identified a number of genes (mainly related to lipid metabolism and immunity) with differential expression between the two morphs. To the best of our knowledge, the only RNA-Seq study so far published that has investigated variation in sexually selected traits in a non-model organism was performed on antler growth in the Sika deer (Yao et al. 2012).

We observed no significant differential gene expression between males of different color morphs or mating strategies, but some of the coloration candidate genes showed tendencies toward such differences (see below). Our small sample size and the large individual variation in gene expression severely limited our power to detect small differences in gene expression between morphs, especially for lowly transcribed genes. A number of recent studies have showed significant gene expression divergence between individuals differing in ecologically important traits, such as disease resistance (Bonneaud et al. 2011) and coloration (Wolf et al. 2010). In a recent study on the cheetah (*Acinonyx jubatus*), gene expression was compared between two sequenced cDNA libraries from skin tissue. One of these was from a black-pigmented spot and the other from an adjacent yellow pigment region. Several genes downstream of the MC1R gene in the melanin synthesis pathway were identified as having higher expression in the black tissue compared with the light one (Hong et al. 2011). Interestingly, one of the most highly expressed genes in our dataset belongs to a gene family (connexins) that have recently been identified as important in color pattern development in zebra fish (Watanabe et al. 2012).

Due to our limited sequencing effort, we could only make robust inferences of gene expression differences in a small fraction of the total number of genes identified in the ruff transcriptome. Only 371 of 6309 identified genes showed evidence of expression in all 11 sampled individuals and out of these, a mere 117 had a mean transcript count of more than 10 reads per individual. With more sequencing (for example using an Illumina Hiseq instrument), future studies will be able to make better estimations of gene expression levels between and within individuals, as well as obtaining expression information from a larger number of genes. For future studies, it will also be preferable to include comparisons between samples from differentially colored feathers from the same individual to control for inter-individual effects.

Several SNP markers showed significant nucleotide divergence between males of different color morphs and mating strategies. Most striking was, perhaps, the large number of hits on feather keratin genes. The keratin gene family has been well characterized in chicken (Presland et al. 1989) and keratin structures are closely associated with both structural (Prum et al. 2009) and melanic (Bonser 1995) coloration. Black ruff feathers commonly show iridescence, which results from structural patterning. The only gene to be identified as having structure between color morphs using all analysis approaches was the Xg blood group. To the best of our knowledge, the Xg gene is not known to function in any coloration pathway. This highly polymorphic gene is positioned on the X chromosome in humans (Cartron and Colin 2001), but is not sex linked in chicken. Because of the limited sampling of individuals and color morphs, the genes identified as significantly structured in our study should only be considered as a list of potential candidates for involvement in ornamental color variation. There are likely to be false positives in these analyses as well as a number of important genes that were not sampled due to the limited sequence coverage of many transcripts. Future studies should try to verify the importance of these, for example using a candidate gene approach.

Many of the transcripts identified as being genetically structured between the two different mating strategies of ruff males (Independents and Satellites) belong to non-annotated genes or sequenced cDNA clones with unknown function. These genes may be specific to birds or are too rapidly evolving to identify the mammalian homologs. Follow-up studies need to verify if these are truly important for mating strategy decisions, and if so what their molecular functions are.

The different approaches taken to identify markers with significant nucleotide structure (GenePop, SAM, LOSITAN) gave only partially overlapping results. In particular, the *F*_ST_ outlier analysis performed using the LOSITAN software (Antao et al. 2008) often identified a larger amount of high-divergence loci compared with the other programs used. It is not unusual to get conflicting results using different outlier detection approaches. Simulations have shown that both type I and type II errors occur for many of the methods routinely used, calling for some caution when interpreting the results (Hohenlohe et al. 2010b; Narum and Hess 2011). The approach taken here, to use multiple software, can, to a certain extent, guard against inferring falsely positive results (Luikart et al. 2003).

We also used a candidate gene approach to investigate variation in known coloration genes in more depth. Previous studies have investigated variation specifically in the MC1R gene involved in the melanin synthesis pathway (MacDougall-Shackleton et al. 2003; Nadeau et al. 2007). However, other types of pigmentation such as genes involved in carotenoid coloration (Wade et al. 2009) have also been specifically targeted. Two recent studies adopted a “multiple candidate gene approach” to investigate several genes involved in avian pigmentation and vision genes, to search for elevated levels of genetic structure (Skoglund and Höglund 2010; Lehtonen et al. 2012). We specifically investigated 28 pre-identified coloration candidate genes. We could not identify any nucleotide sequence variation in any of these, but two (EDNRB and TYR) showed signs of differential expression. The EBNRB gene, which has previously been shown to be involved in mice coat color variation, by regulating melanocyte formation (Cook et al. 2005), was only expressed in black males. In contrast, we were only able to detect expression of the TYR gene, a catalyst in the melanin synthesis pathway (Sato et al. 2007), in individuals with rust color.

Although only half a plate of Roche 454 sequencing was used, we still managed to identify and annotate over six thousand genes expressed in ruff feathers (but note that there was very limited sequence coverage for many of these), which represent about a quarter of all genes annotated from the very well-studied chicken transcriptome, where data are available from a range of different tissues. Almost ten thousand of the chicken genes have been identified as being expressed in skin tissue (Chan et al. 2009), which might be expected to show similar expression patterns as feathers. In the zebra finch (*Taeniopygia guttata*), 6460 genes were identified as being expressed in skin tissue using a similar sized RNA-Seq dataset as presented here. Biological functions related to cytoskeletal structures and cell proliferation were overrepresented in genes primarily expressed in zebra finch skin (Ekblom et al. 2010). Feather pulp was among the sampled tissues analyzed using an RNA-Seq approach in the bobwhite quail (*Colinus virginianus*). In this species, 8825 unique genes were identified and functionally annotated (Rawat et al. 2010).

From our ruff transcriptome sequence data, we were also able to identify more than 800 SNPs and almost 200 microsatellites with flanking sequences. These molecular markers, together with the presented inference of genes important in coloration and mating strategy variation, will provide a valuable resource for further studies of ecology and genetics in this extremely interesting and ecologically well-studied species.

## Data Accessibility

454-sequencing data: NCBI SRA (sequence read archive) database, accession number SRA049313.1.

## Supporting Information

**Appendix A1.** Information about investigated candidate colouration genes (pdf).

**Appendix A2.** SNP information and sequences (xls file).

**Appendix A3.** Full SNP flanking sequences (fasta file).

**Appendix A4.** Information about identified microsatellites, including primer sequences (csv file).
