# Supplementary material for: Gene expression divergence and nucleotide differentiation between males of different color morphs and mating strategies in the ruff
Source: Ecol Evol. 2012 Aug 31;2(10):2485–505. doi: 10.1002/ece3.370 (PMC3492775; doi:10.1002/ece3.370)
Supplement: Supplementary file 1 [file ece30002-2485-SD1.pdf]

Symbol	Gene name	Alternative names	Function	Reference	Chicken Ensembl no.	Chicken chromosome	Expression in Ruff data
AGRP	Agouti related protein	ART; AGRT; ASIP2	This gene encodes an antagonist of the melanocortin-3 and melanocortin-4 receptor in the melanin pathway. It also appears to regulate hypothalamic control of feeding behavior via melanocortin receptor.	Nadeau et al 2007<br>Skoglund & Hoglund 2010	ENSGALP00000039351<br>ENSGALP00000003505	11	No
AIM1	absent in melanoma 1	ST4; CRYBG1; beta-gamma crystallin domain containing 1	One single-nucleotide polymorphism in this gene has clear association with major human populations in terms of skin color.	Nakayama et al 2002	ENSGALP00000024718<br>ENSGALP00000036465	3	Expressed in all individuals. No expression divergence detected.
ASIP	Agouti signaling protein	ASP; AGSW; AGTI; AGTIL; SHEP9	In mice, the agouti gene encodes a paracrine signaling molecule that causes hair follicle melanocytes to synthesize pheomelanin, a yellow pigment, instead of the black or brown pigment, eumelanin. This gene is highly similar to the mouse gene and encodes a secreted protein that may affect the quality of hair pigmentation.	Bonilla et al 2005	ENSGALP00000034003	20	Expressed in one individual (Rust/Black).
ATRN	attractin	Mahogony; MGCA; DPPT-L	Atrn showed effects on pigmentation, testicular vacuolation, and spongiform neurodegeneration in mice. The other isoform is a secreted protein involved in the initial immune cell clustering during inflammatory responses that may regulate the chemotactic activity of chemokines.	Nan et al 2009<br>Gunn et al 1999<br>Cota et al 2008	ENSGALP00000025782	4	Expressed in nine individuals. No expression divergence detected.
BCL2	B-cell CLL/lymphoma 2	Apoptosis regulator Bcl-2; PCKBCL2	Dificiency in this gene in mice induces gray coloration and decrease in the number of lymphocytes.	Nakayama et al 1994	ENSGALP00000036387<br>ENSGALP00000020984	2	No
DCT	Dopachrome tautomerase	TYRP2; dopachrome delta-isomerase, tyrosine-related protein 2; TRP-2; TYRP2; DCT	melanin pathway; Dct activity is 3-fold lower in slaty cells compared with non-agouti black melanocytes, whereas slaty light melanocytes have a surprisingly 28-fold lower Dct activity.	Skoglund & Hoglund 2010<br>Costin et al 2005	ENSGALP00000027266<br>ENSGALP00000035734<br>ENSGALP00000035733	1	No
EDN3	Endothelin 3	ET3; WS4B; HSCR4	The interaction of this endothelin with EDNRB is essential for development of neural crest-derived cell lineages, such as melanocytes and enteric neurons. When Edn3 is driven by the keratin 5 promoter and thereby placed proximal to melanocyte lineage cells, adult mice manifest pigmented skin harboring dermal melanocytes.	Cook et al 2005<br>Garcia et al 2008	ENSGALP00000038270	20	No
EDNRB	Endothelin receptor B	ETB; ETBR; ETRB; HSCR; WS4A; ABCDS	An insertion of retrotransposon in this gene causes piebald coat color in mice.	Cook et al 2005<br>Ohtani et al 2004	ENSGALP00000027287<br>ENSGALP00000035729	1	Expressed in two individuals; both black.
EDNRB2	endothelin receptor B subtype 2	MGC147408	Overexpression of this gene can maintain normal dorsolateral migration of melanoblasts in the absence of EphB2.	Harris 2008	ENSGALP00000038783<br>ENSGALP00000012058<br>ENSGALP00000012059	4	No
ESR1	estrogen receptor 1	ER-alpha; nuclear receptor subfamily 3 group A member 1	Influences skin coloration, sexual development and reproductive function.	Izagirre et al 2006	ENSGALP00000021137	3	No
GNA11	guanine nucleotide binding protein (G protein), alpha 11 (Gq class)	Q71RI7; Dsk7	A new class of dominant dark skin (Dsk) mutations has been linked to this gene.	Van Raamsdonk et al 2004	ENSGALP00000005115	Un	Expressed in one individual (White).
GNAQ	guanine nucleotide binding protein (G protein), q polypeptide	RCJMB04_23b22	A new class of dominant dark skin (Dsk) mutations has been linked to this gene.	Van Raamsdonk et al 2004	ENSGALP00000024446	Z	No
KIT	Mast/stem cell growth factor receptor	v-kit Hardy-Zuckerman 4 feline sarcoma viral oncogene homolog; PBT; SCFR; C-Kit; CD117	There is a close association between sequence polymorphism in this gene and the roan coat color in horses. Mutations in this gene are also associated with gastrointestinal stromal tumors, mast cell disease, acute myelogenous lukemia, and piebaldism.	Cook et al 2005<br>Marklund et al 1999	ENSGALP00000036735<br>ENSGALP00000022531	4	Expressed in one individual (Black).
KITLG	Kit ligand	SCF; Melanocytic mitogen stem cell factor; MGF; mast cell growth factor	A mutation in close proximity to this gene is associated with hair color variation.	Cook et al 2005<br>Sulem et al 2007	ENSGALP00000018246	1	No
MC1R	Melanocortin 1 receptor	E-locus; alpha melanocyte stimulating hormone receptor; MSH-R	A gene in the melanin pathway. Gene mutations that lead to a loss in function are associated with increased pheomelanin production, which leads to lighter skin and hair color. Over 30 variant alleles have been identified which correlate with skin and hair color, providing evidence that this gene is an important component in determining normal human pigment variation.	Nadeau et al 2007<br>Mundy 2005	ENSGALP00000038021	11	No

MC4R	Melanocortin 4 receptor	Glu3; Fatboy	A gene in the melanin pathway. Also associated with sexual activity and obesity.	Rees 2003<br>Ducrest et al 2008	ENSGALP00000021014	2	No
MCHR1	melanin-concentrating hormone receptor 1	SLC1; GPR24; MCH1R	The pigmentary function of αMSH in teleost fish, presumably mediated by MC1R, is functionally antagonized by the action of MCH which binds to this gene.	Logan et al 2003	ENSGALP00000030706	18	No
MITF	microphthalmia-associated transcription factor	cmi9; MI; WS2; WS2A	This gene regulates the differentiation and development of melanocytes retinal pigment epithelium and is also responsible for pigment cell-specific transcription of the melanogenesis enzyme genes.	Cook et al 2005<br>Liu & Fisher 2010	ENSGALP00000012434<br>ENSGALP00000035445	12	Expressed in one individual (Black).
OCA2	oculocutaneous albinism II	pink-eye dilution homolog, mouse; P protein; P; BEY; PED; BEY1; BEY2; BOCA; EYCL; HCL3; EYCL2; EYCL3; SHEP1	An integral membrane protein involved in small molecule transport, specifically tyrosine - a precursor of melanin. Mutations in this gene result in type 2 oculocutaneous albinism.	Toyofuku et al 2002	ENSGALP00000026970	1	Expressed in five individuals. No expression divergence detected.
PAX3	paired box 3	WS1; WS3; CDHS; HUP2	This gene activates expression of Mitf, a transcription factor critical for melanogenesis, while at the same time it competes with Mitf for occupancy of an enhancer required for expression of dopachrome tautomerase, an enzyme that functions in melanin synthesis.	Cook et al 2005<br>Lang et al 2005	ENSGALP00000006282<br>ENSGALP00000008414<br>ENSGALP00000040058	9	No
PLDN	pallidin homolog	PA; PALLID; RCJMB04_25a23; syntaxin 13-interacting protein pallidin; BLOC-1	One of a number of genes that in mice are associated with pigmentation defects and platelet dense granule deficiency.	Falcón-Pérez & Dell'Angelica 2002	ENSGALP00000009199	10	Expressed in one individual (Black).
PMEL	premelanosome protein	Silver; GP100, MMP115; SILV; melanocyte protein Pmel 17; PME17	Critical for the maturation of eumelanosomes (expression of dark pigmentation). Also related to aggressive behaviour in chicken.	Natt et al 2007<br>Kerje et al 2004	ENSGALP00000023540	LGE22C19W28_E50C23	No
POMC	proopiomelanocortin	adrenocorticotropin; beta-lipotropin; alpha-melanocyte stimulating hormone; beta-melanocyte stimulating hormone; beta-endorphin; LPH; MSH; NPP; POC; ACTH; CLIP	Codes for peptides with roles in pain and energy homeostasis, melanocyte stimulation, and immune modulation. Mutations in this gene have been associated with early onset obesity, adrenal insufficiency, and red hair pigmentation.	Rees 2003	ENSGALP00000026742<br>ENSGALP00000035978	3	No
SLC45A2	solute carrier family 45, member 2	membrane-associated transporter protein; MATP	Variants of this gene induces specific inhibition of expression of red pheomelanin in Silver chickens.	Backstrom et al 2010<br>Gunnarsson et al 2007	ENSGALP00000005236	Z	No
SNAI2	snail homolog 2	SLUG; WS2D; SLUGH1	In mice, knockout mutations in this gene result in mice with coat color dilution and white spots.	Cook et al 2005<br>Steingrímsson et al 2006	ENSGALP00000024548	2	Expressed in four individuals. No expression divergence detected.
SOX10	SRY (sex determining region Y) box 10	DOM; WS4; WS2E; cSOX10; Transcription factor SOX-10	This gene encodes a member of the SOX (SRY-related HMG-box) family of transcription factors involved in the regulation of embryonic development and in the determination of the cell fate. It is highly expressed in unpigmented melanocyte precursors but are down-regulated upon differentiation. The Dark brown plumage color in chickens is caused by an 8.3-kb deletion upstream of this gene.	Cook et al 2005<br>Gunnarsson et al 2011	ENSGALP00000020056	1	No
TYR	tyrosinase	oculocutaneous albinism IA; C locus; CMM8; OCA1A; OCAIA; SHEP3	The enzyme encoded by this gene catalyzes the first 2 steps, and at least 1 subsequent step, in the conversion of tyrosine to melanin. Mutations in this gene result in oculocutaneous albinism, and nonpathologic polymorphisms result in skin pigmentation variation.	Skoglund & Hoglund 2010<br>Sato et al 2007	ENSGALP00000035620<br>ENSGALP00000035621<br>ENSGALP00000035622<br>ENSGALP00000027812	1	Expressed in two individuals; both Rust (p=0.0039)
TYRP1	5,6-dihydroxyindole-2-carboxylic acid oxidase	tyrosinase-related protein 1; TRP1; DHICA oxidase; TRP; CAS2; CATB; GP75	This gene encodes a melanosomal enzyme that belongs to the tyrosinase family and plays an important role in the melanin biosynthetic pathway. Defects in this gene are the cause of rufous oculocutaneous albinism and oculocutaneous albinism type III.	Skoglund & Hoglund 2010<br>Backstrom et al 2010<br>Gratten et al 2007	ENSGALP00000037537<br>ENSGALP00000024489	Z	Expressed in three individuals. No expression divergence detected.

Backstrom et al 2010 Evolution 64:3461-3475
Bonilla et al 2005 Human Genetics 166:402-406
Cook et al 2005 Exp Cell Res 308:222-235
Costin et al 2005 Biochem J. 2005 391:249-59.
Cota et al 2008 Genesis. 2008 46:562-73
Ducrest et al 2008 Trends in Ecology & Evolution 23:502-510
Falcón-Pérez & Dell'Angelica 2002 Pigment Cell Res. 15:82-6.
Garcia et al 2008 J Invest Dermatol. 128:131-42
Gratten et al 2007 Proc. R. Soc. B 274:619-626
Gunn et al 1999 Nature. 1999 398:152-6.
Gunnarsson et al 2007 Genetics 175: 867-877
Gunnarsson et al 2011 Pigment Cell & Melanoma Research 24:268–274
Harris 2008 Development. 135:4113-22.
Izagirre et al 2006 Molecular Biology and Evolution 23:1697-1706
Kerje et al . 2004 Genetics 168:1507-1518
Lang et al 2005 Nature 433: 884-887
Liu & Fisher 2010 Pigment Cell Melanoma Res. 23:741-5.
Logan et al 2003 Genomics 81:184-191
Marklund et al 1999 Mammalian Genome 10:283-288,
Mundy 2005 Proc. R. Soc. B 22 August 2005 272:1633-1640
Nadeau et al. 2007 Proc Roy Soc Lond B. 274:1807-1813
Nakayama et al 1994 PNAS 91:3700-3704
Nakayama et al 2002 Journal of Human Genetics 47: 92-94
Nan et al 2009 Int j Cancer 125:909-17
Nätt et al. 2007 Behav Genet. 2007 37:399-407.
Ohtani et al 2004 Exp Anim 53:S67
Rees 2003 Annual Review of Genetics 37:67-90
Sato et al 2007 Poult Sci 86:2126-2133
Skoglund & Hoglund 2010 PLoS ONE 5:e10334
Steingrímsson et al 2006 Developmental Dynamics 235:2401–2411
Sulem et al 2007 Nature Genetics 39:1443-1452
Toyofuku et al 2002 Pigment Cell Res. 15:217-24
Van Raamsdonk et al 2004 Nature Genetics 36: 961-968
