# Supplementary figures and images for: Gene expression divergence and nucleotide differentiation between males of different color morphs and mating strategies in the ruff
Source: Ecol Evol. 2012 Aug 31;2(10):2485–505. doi: 10.1002/ece3.370 (PMC3492775; doi:10.1002/ece3.370)

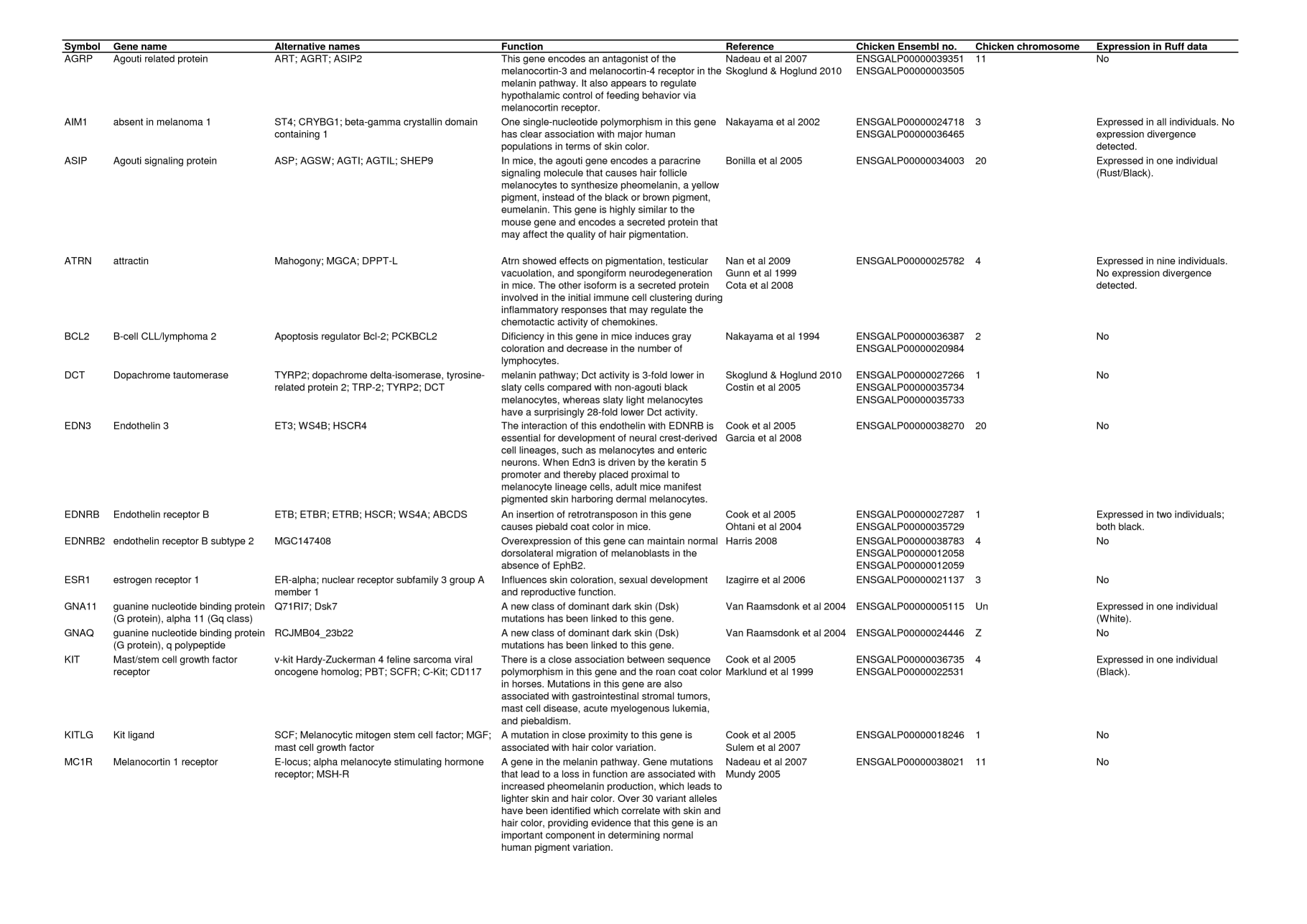

Supplement: Supplementary file 2 [file ece30002-2485-SD5.png]
